# Classification and prediction of *Klebsiella pneumoniae* strains with different MLST allelic profiles *via* SERS spectral analysis

**DOI:** 10.7717/peerj.16161

**Published:** 2023-09-25

**Authors:** Li-Yan Zhang, Benshun Tian, Yuan-Hong Huang, Bin Gu, Pei Ju, Yanfei Luo, Jiawei Tang, Liang Wang

**Affiliations:** 1 Laboratory Medicine, Ganzhou Municipal Hospital, Guangdong Provincial People’s Hospital Ganzhou Hospital, Ganzhou, Guangdong Province, China; 2 Laboratory Medicine, Guangdong Provincial People’s Hospital, Guangdong Academy of Medical Sciences, Guangzhou, Guangdong Province, China; 3 School of Life Sciences, Xuzhou Medical University, Xuzhou, Jiangsu Province, China

**Keywords:** *Klebsiella pneumoniae*, MLST, SERS, Hospital-acquired infection, Transmission

## Abstract

The Gram-negative non-motile *Klebsiella pneuomoniae* is currently a major cause of hospital-acquired (HA) and community-acquired (CA) infections, leading to great public health concern globally, while rapid identification and accurate tracing of the pathogenic bacterium is essential in facilitating monitoring and controlling of *K. pneumoniae* outbreak and dissemination. Multi-locus sequence typing (MLST) is a commonly used typing approach with low cost that is able to distinguish bacterial isolates based on the allelic profiles of several housekeeping genes, despite low resolution and labor intensity of the method. Core-genome MLST scheme (cgMLST) is recently proposed to sub-type and monitor outbreaks of bacterial strains with high resolution and reliability, which uses hundreds or thousands of genes conserved in all or most members of the species. However, the method is complex and requires whole genome sequencing of bacterial strains with high costs. Therefore, it is urgently needed to develop novel methods with high resolution and low cost for bacterial typing. Surface enhanced Raman spectroscopy (SERS) is a rapid, sensitive and cheap method for bacterial identification. Previous studies confirmed that classification and prediction of bacterial strains *via* SERS spectral analysis correlated well with MLST typing results. However, there is currently no similar comparative analysis in *K. pneumoniae* strains. In this pilot study, 16 *K. pneumoniae* strains with different sequencing typings (STs) were selected and a phylogenetic tree was constructed based on core genome analysis. SERS spectra (N = 45/each strain) were generated for all the *K. pneumoniae* strains, which were then comparatively classified and predicted *via* six representative machine learning (ML) algorithms. According to the results, SERS technique coupled with the ML algorithm support vector machine (SVM) could achieve the highest accuracy (5-Fold Cross Validation = 100%) in terms of differentiating and predicting all the *K. pneumoniae* strains that were consistent to corresponding MLSTs. In sum, we show in this pilot study that the SERS-SVM based method is able to accurately predict *K. pneumoniae* MLST types, which has the application potential in clinical settings for tracing dissemination and controlling outbreak of *K. pneumoniae* in hospitals and communities with low costs and high rapidity.

## Introduction

The Gram-negative non-motile bacterium *Klebsiella pneumoniae* was first isolated from a pneumonia patient in 1875 by Edwin Klebs and further characterized by Carl Friedlander in 1882 (Köhler & Mochmann, 1987). Although *K. pneumoniae* is an opportunistic pathogen, the bacterium is able to cause infection in multiple sites in human beings such as lungs, bloodstream, and liver, *etc*., leading to pneumonia, sepsis, and liver abscess (Ballén et al., 2021; Heiden et al., 2020). Due to the increased antibiotic resistance of the pathogen, multidrug resistant *K. pneumoniae* could cause extremely difficult-to-treat infections due to limited therapeutic options (Liu et al., 2022). In addition, previous studies show that the bacterium is mainly responsible for hospital-acquired (HA) and community-acquired (CA) infections, primarily among immunocompromised patients, the elderly, and the newborns (Meng et al., 2019). Therefore, it is important to rapidly and accurately identify *K. pneumoniae* strains with genotyping methods so as to tracking the transmission routes of the pathogen in hospital and/or community settings, which will facilitate the prevention and control of the bacterial pathogen.

Bacterial genotyping is mainly used in microbiology for epidemiological surveillance, which is important to identify bacterial outbreaks and is able to track the origin and spreading of infectious agents (Ochoa-Díaz, Daza-Giovannetty & Gómez-Camargo, 2018). Currently, methods of bacterial genotyping include pulsed field gel electrophoresis (PFGE), multiple-locus variable number tandem repeat analysis (MLVA), multi-locus sequence typing (MLST), core-genome MLST (cgMLST), and core single nucleotide polymorphism (coreSNP), *etc*. (Gona et al., 2020; Zhou et al., 2017), among which PFGE, core-genome MLST (cgMLST), and core single nucleotide polymorphism (coreSNP) are the three most frequently used methods for the typing of *K. pneumoniae* strains (Gona et al., 2020). As a traditional but standard method, MLST was first proposed in 1998 and has been widely applied in characterizing bacterial strains *via* house-keeping genes, through which distinct alleles for each housekeeping gene are assigned and all alleles from all the chosen house-keeping genes are combined together to define the allelic profile that is also known as sequence type (ST) (Maiden et al., 2013). MLST only uses a short list of housekeeping genes and the analytical results have low resolution, while cgMLST is a novel molecular typing method with high-resolution that is based on whole genomic sequencing, which has high accuracy in bacterial typing and tracing and is gaining more acceptance in sequence typing analysis (Yan et al., 2021). However, these typing methods suffer their own limitations such as complex procedures, high costs, and low discrimination capacity, *etc*., which greatly hinders their practical use in real-world settings like clinical laboratory diagnosis. Therefore, novel methods are urgently needed to type and track the transmission of bacterial pathogens rapidly and accurately.

Surface enhanced Raman spectroscopy (SERS) is a highly sensitive and non-invasive method that has been employed for identifying bacterial species, antibiotic resistance, and virulence phenotypes through the combination of computational methods such as clustering algorithms and machine learning methods (Liu et al., 2022; Lyu et al., 2023; Usman et al., 2022; Wang et al., 2021). Therefore, the technique holds the potential in discriminating bacterial strains with different sequence typing. A previous study has already showed that SERS spectral analysis had advantages over traditional genotyping methods for epidemiological surveillance of bacterial infections in terms of rapidity, automation and reliability (Lu et al., 2013). A pilot study also confirmed that the label-free SERS technique could identify antibiotic resistant isolates of three MLST-predefined living *Escherichia coli* groups (Cheong et al., 2017). However, there is, so far, limited studies focus on using label-free SERS technique for bacterial molecular typing in *Klebsiella* genus. A couple of studies provided preliminary but conflict results when comparing Raman spectroscopy with molecular typing for bacterial pathogens from the genus *Klebsiella*, which suggests that further studies are needed to compare the two methods (Dieckmann et al., 2016; Overdevest et al., 2014).

**Figure 1:**
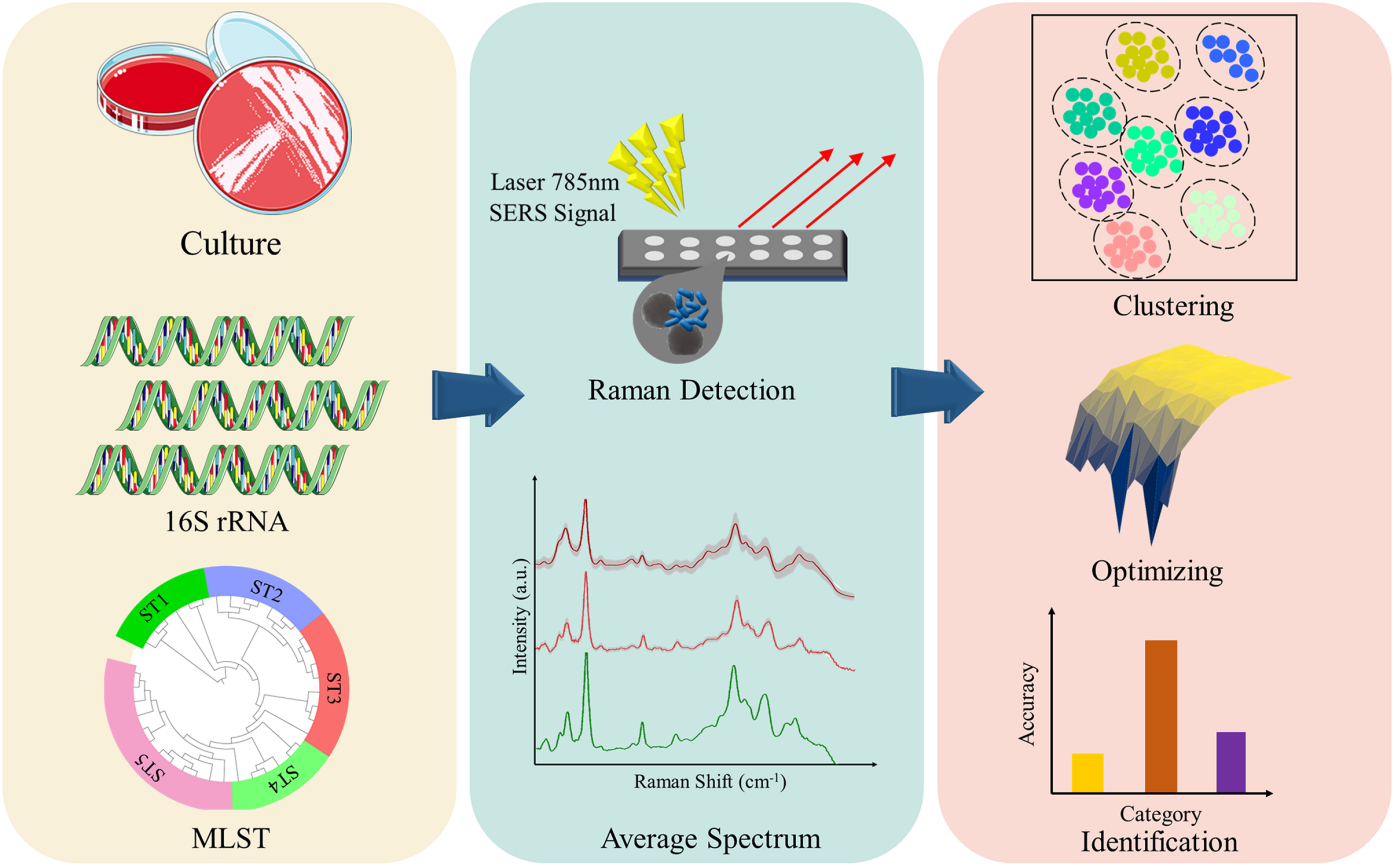
Schematic illustration of the experimental and computational workflow of this study, which involves bacterial culture, genome sequencing, sequence typing, SERS spectral collection, and computational analysis of SERS spectra generated from *K. pneumoniae* strains with different STs.

In order to elucidate the capacity of SERS technique in discriminating and predicting *K. pneumoniae* strains with different STs, we selected 16 *Klebsiella pneumoniae* strains with distinct STs that were isolated from clinical samples. SERS technique was then applied to these *K. pneumoniae* strains to generate average SERS spectrum for each ST type. Classification analysis *via* Orthogonal Partial Least Squares-Discriminant Analysis (OPLS-DA) showed that SERS spectra belonging to different *K. pneumoniae* strains could cluster into different groups, while machine learning analysis confirmed the support vector machine (SVM) algorithm can achieve accurate prediction of *K. pneumoniae* strains with different STs, which is consistent with MLST analysis. Taken together, in this pilot study, we show that the SERS-SVM based method is able to accurately recognize *K. pneumoniae* MLST types for the first time, which has the application potential in clinical settings for tracing dissemination and controlling outbreak of *K. pneumoniae* strains in hospitals and communities with low costs, short time and high accuracy (Fig 1).

## Methods and materials

### Collection of *K. pneumoniae* strains

*K. pneumoniae* strains were obtained from the Clinical Microbiology Laboratory at Guangdong Provincial People’s Hospital, Guangzhou, Guangdong Province, China. All the bacterial strains were grown overnight in commercial Luria Bertani (LB) liquid medium to the exponential growth phase and bacterial cells were collected by centrifugation at 4,500 rpm for 8 min followed by keeping the pellet and discarding the supernatant. The pellet was re-suspended in 2 mL of distilled deionized water (ddH_2_O). Bacterial concentration was determined by plate-counting test performed on blood agar plates incubated at 37 °C for 24 h wherever needed. All experiments in contact with bacteria were sterilized *via* autoclaving at 121 °C for 30 min.

### Whole genome sequencing of *K. pneumoniae* strains

The Illumina MiSeq Instrument was used for paired-end (PE) sequencing, and the sequencing mode was set to be PE300. All the reads obtained by sequencing were evaluated *via* FastQC Software (version: 0.11.9; https://www.bioinformatics.babraham.ac.uk/projects/fastqc/) for sequencing quality control, and then the software Trimmomatic (version: 0.39) was used to remove sequences with a probability of higher than 1% of wrong bases, sequences rich in adapters and sequences with too many N bases (Bolger, Lohse & Usadel, 2014). Finally, the SPAdes software (version: 3.1.2) was used for reads assembly, and all the contigs less than 200 bp were removed, and finally assembled whole genomes of *K. pneumoniae* strains with high-quality were obtained for further analysis (Bankevich et al., 2012). All the 16 *K. pneumoniae* genome assembled from raw reads have been submitted to NCBI server (BioProject ID: PRJNA960686).

### Core-genome and phylogenetic analyses

The genome annotation software PROKKA (Version: 1.13) was used to annotate the whole genome sequences of 16 *K. pneumoniae* strains (Seemann, 2014). Then, Roary (Version: 3.13.0), a software enabling rapid large-scale prokaryotic pan-genome analysis, was used for core genome analysis with a minimum percentage identity of 95%, and a total of 3,364 core genes were generated by analyzing the 16 *K. pneumoniae* strains (Page et al., 2015). FastTree (Version: 2.1.12) was then run with “*-nt”* and “*-gtr”* settings to produce a phylogenetic tree in Newick format (Price, Dehal & Arkin, 2009). Finally, the Newick tree file produced by FastTree was imported into the webserver interactive Tree of Life (iTOL6) for phylogenetic tree visualization (Letunic & Bork, 2021).

### Multi-locus sequence typing (MLST)

Based on the whole genome sequencing result, MLST software (version: 2.23.0) was used to scan the overlapping parts of the seven conserved housekeeping genes (*gapA, infB, mdh, pgi, phoE, rpoB, tonB*) within the assembled genome sequences of 16 *K. pneumoniae* strains. Through comparing the seven housekeeping genes in all the *K. pneumoniae* strains, sequence typing was determined. The seven conserved housekeeping genes are provided by the Public Databases for Molecular Typing and Microbial Genome Diversity (PubMLST).

### Preparation of silver nanoparticles (AgNPs)

The preparation of silver nanoparticles (AgNPs) was based on a classical and facile chemical reduction method, which was recorded in details in previous studies (Liu et al., 2023; Tang et al., 2022). All the AgNPs used in this study were synthesized by ourselves in the lab at Guangzhou Provincial People’s Hospital, Guangzhou, China. For the characterization of dimensions and morphology of the AgNPs, please refer to our recent publication (Lyu et al., 2023).

### SERS spectral generation

SERS spectra were collected by using the InVia Reflex Confocal Raman Microscope (Renishaw, Wotton-under-Edge, UK). The Raman spectroscope was equipped with a 785 nm diode laser, achieving a spectral resolution of less 1 cm^−1^. A bacteria sample (10 μl) was mixed with 10 μl of AgNPs and then incubated for 15 min to make sliver nanoparticles sufficiently interacted with the sample before dropping the mixture on silicon wafer. The wavelength of the instrument was calibrated automatically using an interior silicon wafer plus manual adjustment of external silicon wafer by setting the silicon peak at 520 cm^–1^. Bacterial samples were excited with a near infrared 785 nm diode laser in a range of 500–1,800 cm^−1^. The Raman excitation light was focused onto the sample using a 50× objective lens, with a laser power of 150 mW. To ensure the stability and reproducibility of the results, a fixed integration time of 20 s per spectrum was implemented. For each *K. pneumoniae* strain, a total of forty-five spectra were collected under controlled conditions of constant room temperature, guaranteeing the consistency of spectral acquisition for each sample (Bashir et al., 2021).

### Average SERS spectra and deconvolution analysis

Average intensity of all replicated Raman spectra (N = 45) at each Raman shift was calculated to generate an average SERS spectrum for one ST typing strain of *K. pneumoniae*, and the spectral standard deviation (SD) was calculated and visualized in the average SERS spectrum for indicating the stability of the experimental data. The software Origin (Version 2019b; OriginLab, Northampton, MA, USA) was used to plot average Raman spectra, in which the shaded error band part represented SD. The wider the error band, the worse the reproducibility. Spectral characteristic peaks were generated by using LabSpec6 (HORIBA Scientific, Kyoto, Japan). In specificity, GaussLoren function was used for fitting peaks with parameters set to Level = 15%, Size = 20. All the identified characteristic peaks were shown in the form of dot plot. Biological meanings of all the characteristic peaks were sourced from literature. Due to the high similarity of the different ST average Raman spectra, in order to explore the differences between different spectra, spectral deconvolution was conducted to process the average Raman spectrum for each ST classification. Specifically, the function of *fit peaks pro* in Origin software was used to fit characteristic peaks, and the function *Vogit* as the convolution form of Gaussian and Lorentzian functions was used to generate deconvolution sub-band for each average SERS spectrum.

### SERS spectral clustering analysis

Raman spectral clustering aims to divide spectral dataset into different clusters according to a specific rule. Unsupervised learning algorithms like principal component analysis (PCA) are often used to analyze spectra by calling *PCA* function in sci-kit learn (version 0.21.3) data analysis library (Ayala et al., 2018; Bashir et al., 2021). In particular, *fit_transform* method was used to fit SERS spectra for different ST typing *K. pneumoniae* strains, and the top two principal components PC1 and PC2 with the largest contribution were selected to describe the overall characteristics of SERS spectra. However, due to the mild differences between SERS spectra of different ST types, the clustering effects were not good due to interfering factors that were not relevant to the grouping information. Therefore, Orthogonal Partial Least Squares Discriminant Analysis (OPLS-DA) was used to avoid the influence of interference factors in SERS spectral data on the classification results. Specifically, the OPLS-DA function from multivariate statistical analysis software SIMCA (version 13.0, 32 bit) was applied to automatically fit all SERS data from different ST types, which separated the data that were not relevant to the classification information from the data matrix. The results of PCA and OPLS-DA clustering methods were shown in scatter plots, and SERS spectra from *K. pneumoniae* strains with ST types were marked with black dashed circles.

### Supervised machine learning analysis of SERS spectra

#### Division of SERS spectra dataset

To achieve rapid and accurate identification of *K. pneumoniae* strains with different STs, we compared the prediction performance of six machine learning algorithms, that is, Adaptive boosting (AdaBoost), Decision Tree (DT), eXtreme Gradient Boosting (XGB), Quadratic Discriminant Analysis (QDA), Random Forest (RF), and SVM on SERS spectra. Before conducting data analysis, in order to optimize model training efficiency, we utilized the *train_test_split* function to reasonably divide the dataset into training, validation, and test sets with a ratio of 6:2:2. The training set was employed for constructing the machine learning model, while the validation set played a pivotal role in assessing the model’s development process and providing the unbiased estimations. The test set, exclusively dedicated to evaluating the performance of the final trained model, remained separated from the model construction process (Tang et al., 2022).

#### Model parameter optimization

In order to determine the optimal performance of the final identification model among different models and within all parameter ranges of the model itself, GridSearch was used to determine the optimal combination of model parameters, all hyperparameter ranges for each model pre-defined in the program (Table S1). Specifically, *GridSearchCV* function was used to optimize the hyperparameter combination, and the *cv* parameter was set to 5, which means that five times of cross validation would be performed. The hyperparameter combination with the highest average score was taken as the best for the final model training. We recorded all the parameter combinations for each model and visualized the gradient model scores.

#### Model performance evaluation

Quantitative evaluation of model effectiveness is key to determine model performance. In this study, seven evaluation indexes including Accuracy, Precision, Recall, F1-score, fitting time, area under the curve (AUC) and five-fold cross validation were used to evaluate the model performance. For evaluating the predication capacity of machine learning models, there are four main categories: (1) True Positive (TP); (2) False Negative (FN); (3) False Positive (FP); and (4) True Negative (TN). The accuracy score describes the proportion of the predicted correct samples in the total number of samples by calling the *accuracy_score* function. The calculation formula is as follows:

$Accuracy = \; \displaystyle{{TP + TN} \over {TP + TN + FP + FN}}$

In order to avoid the unbalance in dataset splitting and due to the unmeasurable of real predictive ability of the model, Precision and Recall were used for evaluation. Precision was calculated by calling *precision_score* (average = ‘micro’) function to indicate how many of the predicted true samples are true. Recall was calculated by calling *recall_score* (average = ‘macro’) to indicate how many true values are recognized by the model in the actual dataset. The formula for calculating these two indexes is as follows:

$Precision = \; \displaystyle{{TP} \over {TP + FN}},\; Recall = \; \displaystyle{{TP} \over {TP + FP}}$

Precision and Recall are a pair of mutually restrictive metrics, in order to comprehensively consider the factors of the two metrics, F1-score is used as the weighted harmonic average of Precision and Recall. The *f1_score* (average = ‘weighted’) function was called to measure the model’s ability to find true value. The formula is as follows:

$F1\; = \displaystyle{{2*Precision*Recall} \over {Precision + Recall}}\;$

The AUC value is the area under the curve of the operating characteristic curve. Different from the above metrics, this metrics does not depend on the selection of threshold. The larger the area under the curve is, the better the model effect will be. In this study *roc_auc_score* function was used to calculate the value of AUC. The calculation formula is:

$AUC = \displaystyle{1 \over 2}\mathop \sum \limits_{i\,=\,1}^{n - 1} \left( {TP{R_{i + 1}} + TP{R_i}} \right)\left( {FP{R_{i + 1}} - FP{R_i}} \right)$

*n* represents the total number of points on the ROC curve, each point on the curve represents the classification result of a particular classifier, the coordinates of the last point on the ROC curve are denoted as (FPR_n_, TPR_n_), where FPR_n_ is the False Positive Rate and TPR_n_ is the True Positive Rate at that point (Wang et al., 2022).

Considering the similar prediction performance of different machine learning models, in order to improve the efficiency of model identification and reduce computing costs, we compared the model fitting time on the same dataset, and *time* function was used to record the start time and end time of the model training. The less the time, the lower the computing resources consumed by the model. The calculation formula is:

$Times = Tim{e_{start}} - Tim{e_{end}}$

## Results

### Whole genome sequencing and Core-/Pan-genome analysis

#### Whole genome sequencing

General features of the 16 *K. pneumoniae* genomes are presented in Table 1, which were obtained by integrating genome assembly and annotation results. Genome sizes range from 5.32 to 6.23 Mbps. The number of predicted protein coding sequences (CDSs) in the 16 isolated varied from 4,977 (Strain ID: 2470) to 5,900 (Strain ID: 2497). The overall GC content in these strains ranges from 56.70% to 57.66% and remains relatively consistent among different isolates. All strains have a single tmRNA coding gene. There is a slight variation in the number of ribosome RNA (rRNA) and transfer RNA (tRNA) coding genes among the strains varies, but without significant differences (Wang et al., 2019).

**Table 1:** Basic information of genome sequencing, assembly, and annotation data for the 16 *K. pneumoniae* strains.

Strain ID	Contigs	Bases	CDS	tRNA	rRNA	tmRNA	GC content (%)
1476	202	5,429,699	5,004	83	11	1	57.66
2109	690	5,951,243	5,431	85	11	1	57.47
2130	179	5,634,669	5,255	84	10	1	57.09
2260	98	5,447,909	5,029	85	12	1	57.25
2369	149	5,690,453	5,379	82	12	1	57.01
2424	507	5,745,670	5,254	82	14	1	56.71
2443	245	5,501,232	5,083	84	12	1	57.25
2467	135	5,607,898	5,217	85	16	1	57.05
2468	150	5,534,833	5,100	79	11	1	57.27
2470	57	5,391,662	4,977	79	10	1	57.28
2478	201	5,962,258	5,505	85	12	1	56.67
2481	223	5,921,972	5,531	83	19	1	56.92
2497	222	6,227,838	5,900	89	12	1	56.70
2500	74	5,321,704	4,886	82	13	1	57.45
2501	105	5,364,821	5,021	83	13	1	57.38
2503	226	5,749,244	5,437	80	14	1	57.10

#### Core-/Pan-genome analysis

**Figure 2:**
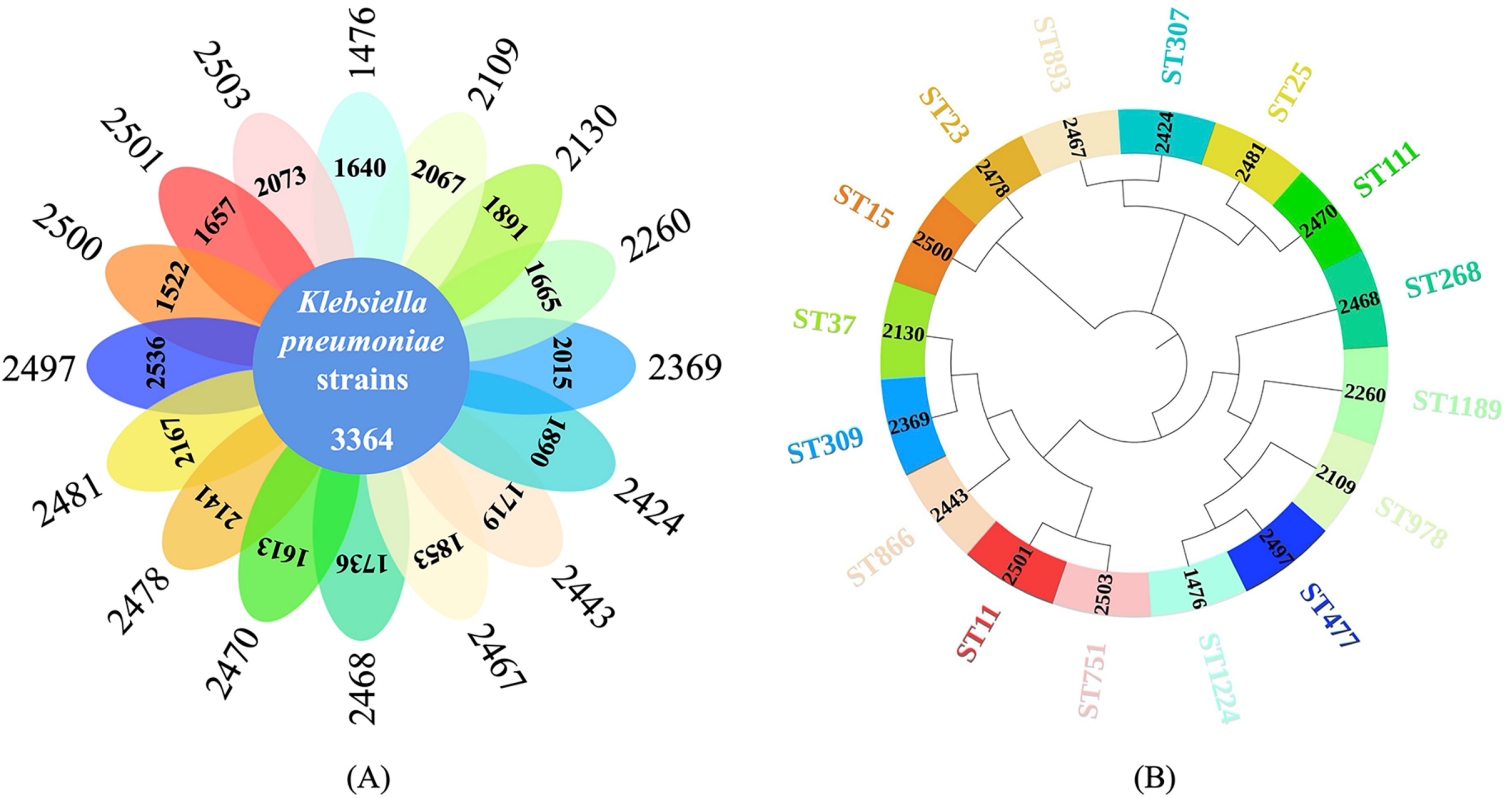
Core-genome and pan-genome analyses of *K. pneumoniae* strains with different STs. (A) Venn diagram of shared and unique CDSs among 16 *K. pneumoniae* strains. (B) Phylogenetic tree constructed *via* core genome analysis of 16 *K. pneumoniae* strains. Computational MLST typing results were labelled in the exterior circle of the phylogenetic tree adjacent to each *K. pneumoniae* strain ID in the interior circle, accordingly.

The total pan-genome analysis for the 16 *K. pneumoniae* strains contain 12,523 coding sequences (CDSs), among which 3,364 (26.86% of total CDSs) were considered as core genes across all 16 strains while 9,159 (73.14% of total CDSs) constituted the accessory fractions, which were unique to each *K. pneumoniae* genome, respectively. The *K. pneumoniae* strain (strain ID: 2500) has the lowest number of the unique genes (1,522 CDSs) while the *K. pneumoniae* strain (strain ID: 2497) has the highest number of unique genes (2,536 CDSs). A Venn diagram was plotted to show the core-genome and pan-genome analysis for all the studied *K. pneumoniae* strains (Fig. 2A) while a phylogenetics tree based on core-genome sequences were generated and visualized (Fig. 2B). MLST was computationally performed based on whole genome sequences, which were labelled on the exterior circle of the phylogenetic tree, indicating the phylogenetic relationship between sequence types.

#### Analysis of average and deconvoluted SERS spectra

**Figure 3:**
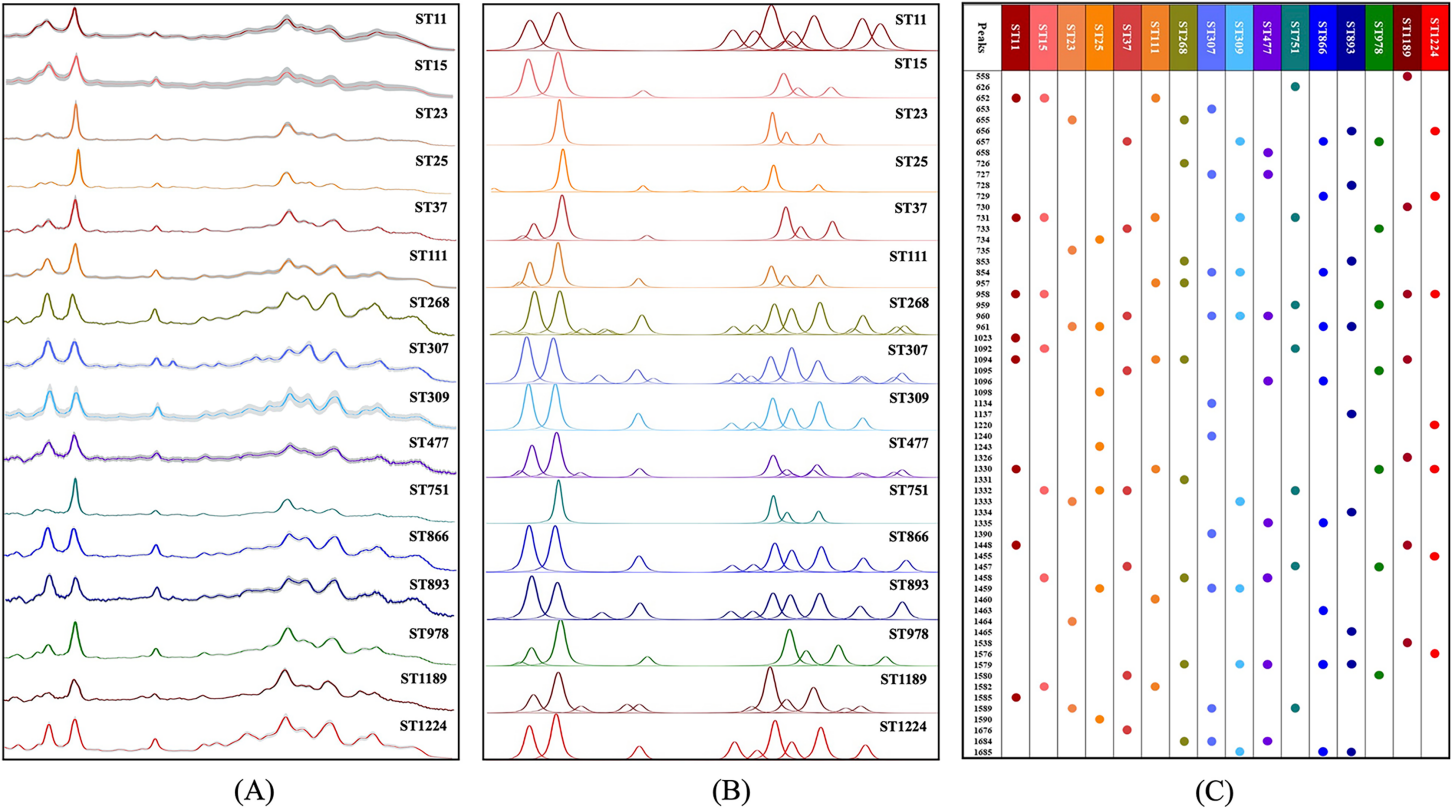
Average and deconvoluted SERS spectra together with characteristic spectral speaks for each *K. pneumoniae* strain with a unique sequence typing. (A) Average SERS spectra of 16 *K. pneumoniae* strains. (B) Deconvoluted SERS spectral bands. X-axis represents Raman shifts in the range of 530–1,800 cm^−1^, while Y-axis represents the relative Raman intensity. (C) Dot matrix indicating the distribution of characteristic peaks for SERS spectra of *K. pneumoniae* strains.

An average Raman spectrum was used to reflect the overall distribution trend of SERS signal intensities for a single *K. pneumoniae* ST type. We calculated the average signal intensities of specific ST type at each Raman shift to generate the average Raman spectrum (Fig. 3A). The Raman signal standard error of each ST type was calculated to describe the degree of reproducibility of the SERS signal. The narrower the error band, the better the spectral reproducibility. Due to the high similarity of partial average spectra, such as ST268 and ST866, it is difficult to see the difference between the two spectra by naked eyes only. Therefore, spectral deconvolution was conducted to fit each spectral characteristic peaks (Fig. 3B). The distribution and intensity changes of spectral characteristic peaks were comprehensively considered. It can be seen that the SERS spectral deconvolution curves of ST268 and ST866 differ obviously in different Raman shift ranges. For example, in the range of 800–900 cm^−1^ and 1,650–1,750 cm^−1^, ST268 has four difference deconvolution peaks than ST866 (790 cm^−1^, 850 cm^−1^, 1,675 cm^−1^, and 1,695 cm^−1^). In addition, we present the fitted spectral peaks in the form of dot matrix (Fig. 3C), and the metabolites corresponding to the peaks of 16 *K. pneumoniae* ST types are sourced from literature and summarized in Table S2.

#### Clustering analysis of *K. pneumoniae* SERS spectra

**Figure 4:**
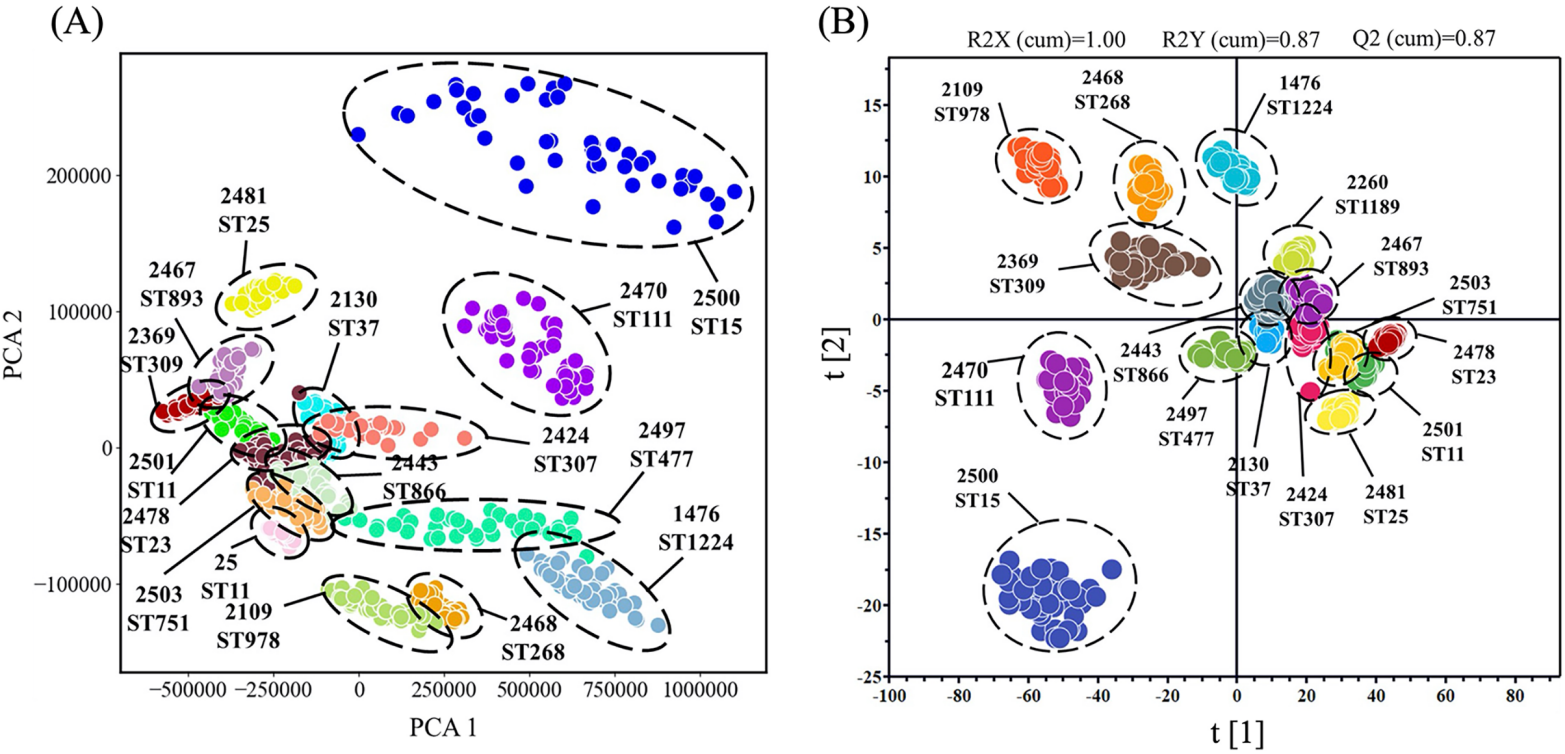
Clustering analysis of SERS spectra of *K. pneumoniae* strains with different STs through the (A) PCA algorithm and the (B) OPLS-DA algorithm. The results were visualized in scatterplot. All the *K. pneumoniae* strains were labelled with both unique Strain ID and sequencing typing.

To provide better insight into the SERS spectral analysis of *K. pneumoniae* strains with different ST types, we firstly used the unsupervised machine learning algorithm PCA to observe the natural clustering trends between different ST types. The results showed that the clustering of SERS spectra for the same ST type was discrete with overlapping among different ST types. In addition, the PCA method could not quantitatively evaluate the clustering results. Therefore, we used the supervised learning algorithm OPLS-DA as an alternative to analyze the SERS spectral of different ST types of *K. pneumoniae* strains. This method was used to weaken the spectral fluctuation of the same ST classification and maximize the difference among the 16 ST types. According the clustering result as shown in Fig. 4, spectral sample points of different ST types were clustered into different clusters, and the score of three performance evaluation indices were R2X (cum) = 1.00, R2Y (cum) = 0.87 and Q2 (cum) = 0.87, indicating that the OPLS-DA algorithm could better distinguish SERS spectral data of different ST types into separated groups. Through the clustering analysis *via* OPLS-DA, it was also revealed that SERS spectra of *K. pneumoniae* with different ST types were separable *via* computational methods, suggesting the intrinsic spectral differences among these strains. However, clustering analysis cannot provide prediction results. Therefore, when clustering new unknown SERS spectral data, the clustering algorithm needs to re-calculate the distribution of each sample point, which cannot quickly classify new samples.

### Machine learning analysis of *K. pneumoniae* SERS spectra

#### Parameter optimization

**Figure 5:**
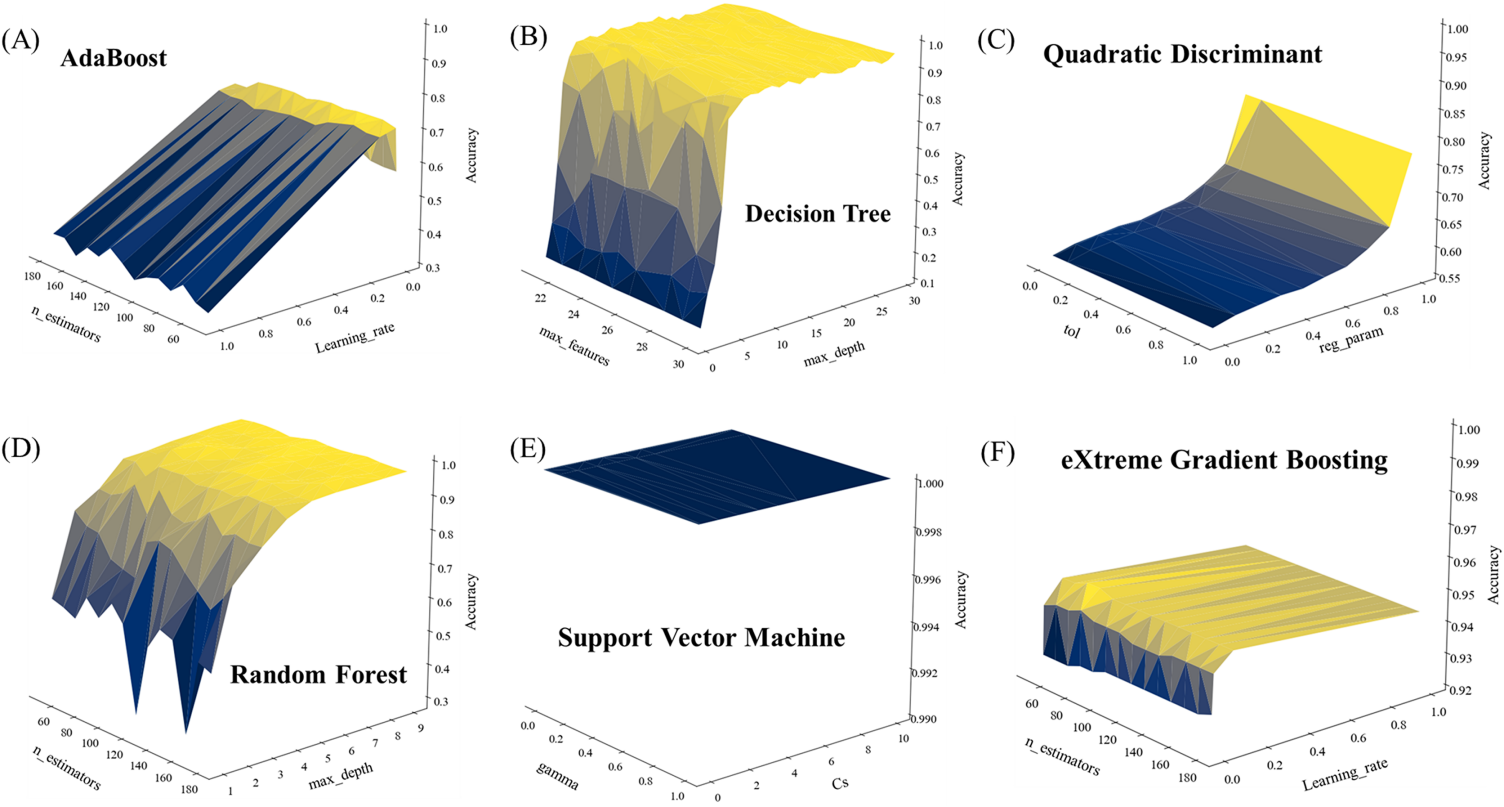
Parameter optimization of six supervised machine learning algorithms used in this study. (A) AdaBoost. (B) Decision tree. (C) Quadratic discriminant. (D) Random forest. (E) Support vector machine. (F) Extreme gradient boosting.

Unlike clustering analysis that cannot provide specific labels for clustered samples, supervised machine learning analysis is able to generate prediction results with specific labels based on trained models (Cunningham, Cord & Delany, 2008). However, different prediction results could be generated with different combinations of hyperparameters, which emphasizes the importance of parameter optimization during modeling training process (Lameski et al., 2015). In this study, the *GridSearch* function was used to obtain the best combination of hyperparameters. According to the *GridSearch* gradient plots (Fig. 5), the accuracy score of SVM in all parameter combinations is 1 (Fig. 5E), indicating that SVM algorithm has excellent analytical ability in small samples of high dimensional data, which is consistent with previous studies (Cheng et al., 2020). DT, RF and XGB algorithms also show good analytical ability (Figs. 5B, 5D and 5F), all quickly reaching to an accuracy score of more than 0.95 within a few parameter ranges, and remaining stable in the majority of parameter combinations. As for the AdaBoost and QDA algorithms, neither of the two algorithms scored above 0.8 for all parameter combinations, indicating that these algorithms need more computing resources.

#### Comparison of supervised machine learning algorithms

In this study, we compared the performance of six supervised machine learning algorithms, and explored their ability in identifying ST types by analyzing SERS signal data from 16 *K. pneumoniae*. Seven machine learning evaluation metrics were used to evaluate different models. Computational results were shown in Table 2, according to which the SVM model achieves the best performance among all algorithms. All score indices for SVM are 100%, and the training time of the model is relatively short (Times = 0.50 s), indicating that the SVM model can accurately and efficiently identify different ST types. RF, DT and XGB also achieved greater than 90% identification accuracy. The fitting time of XGB model was 21.40 s, which consumed the highest amount of computational resource among all algorithms. It is worth noting that the fitting time of the DT model is only 0.01 s, and the identification accuracy was 96.53%, while the five-fold cross validation score was 94.84% with a slight overfitting of the model, which indicated that DT algorithm was a fast identification method of *K. pneumoniae* ST types. In contract, the QDA and AdaBoost algorithms did not achieve good results in the data of this study. The accuracy value of AdaBoost was only 59.03%, and the five-fold CV score was 70.90%, indicating that the model was underfitting and the parameter range should be further expanded.

**Table 2:** Performance comparison of six supervised machine learning algorithms on the prediction of *K. pneumoniae* strains with distinct STs based on SERS spectral analysis.

Algorithm	ACC	Precision	Recall	F1	5-Fold CV	AUC	Time (s)
**SVM**	100.00%	100.00%	100.00%	100.00%	100.00%	100.00%	0.50
**RF**	97.92%	97.92%	98.30%	97.88%	97.04%	98.08%	2.60
**DT**	96.53%	96.53%	96.16%	96.46%	94.98%	96.96%	0.01
**XGB**	93.75%	93.75%	94.16%	93.73%	94.30%	93.67%	21.40
**QDA**	76.39%	76.39%	76.96%	74.47%	76.81%	75.54%	0.09
**AdaB**	59.03%	59.03%	64.61%	52.56%	70.90%	59.00%	5.38

**Figure 6:**
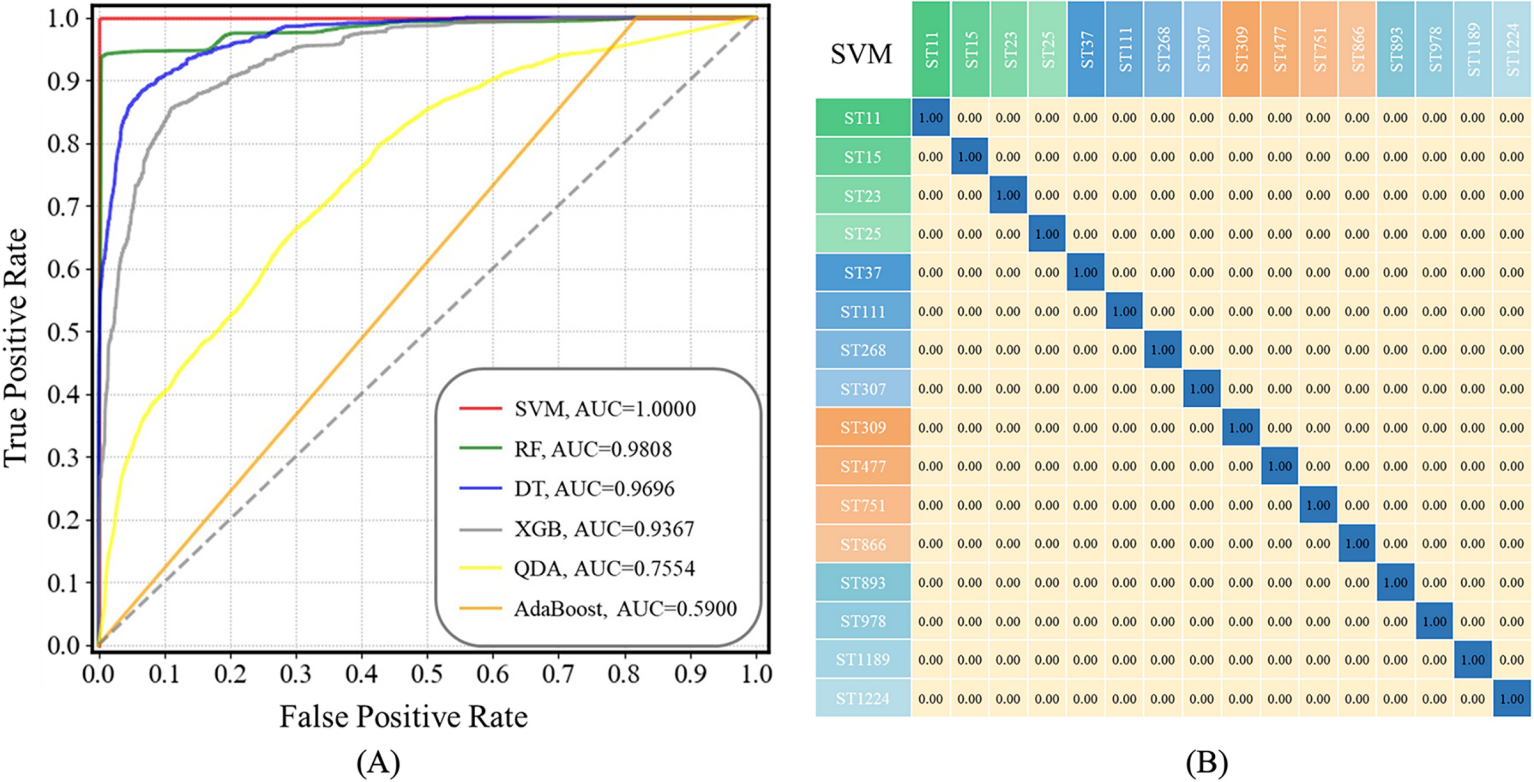
ROC curves for the six machine learning algorithms and the confusion matrix of SVM algorithm when applied to the SERS spectra of *K. pneumoniae* strains with different STs. (A) ROC curve. According to the comparison, SVM achieved the best performance with area under curve (AUC) = 1.00 than all other algorithms. (B) Confusion matrix. The percentages in the confusion matrix stand for the correctly classified (diagonal) or mis-classified (off-diagonal) spectra, respectively.

ROC curves compare the sensitivity and specificity of supervised machine learning methods across a range of values for their predictive capacities, while AUC means overall accuracies in distinguishing data samples (Liu et al., 2023). As for confusion matrix, it is a table summarizing classification results of a supervised machine learning algorithm based on the true class and predicted class (Liu et al., 2022; Wang et al., 2022). In this study, both ROC curves for all the prediction models and a confusion matrix for the optimal prediction model SVM were present in Fig. 6. The x-axis represents specificity (false positive rate, FPR) and the y-axis represents sensitivity (true positive rate, TPR) in ROC curves. It could be seen that the AUC value of the SVM model is equal to 1.00, suggesting that the SVM model had highest specificity and sensitivity during strain prediction (Fig. 6A). As for the confusion matrix, it showed specific performance of the SVM model on the test dataset, according to which, the identification accuracy of the SVM model for *K. pneumoniae* STs was very high, indicating that the optimized SVM model could accurately recognize *K. pneumoniae* STs with very low error rates based on SERS spectral analysis.

## Discussion

*K. pneumoniae* is a common cause of nosocomial and community-acquired infections (Dieckmann et al., 2016). Classification and prediction of *K. pneumoniae* strains is crucial to determine the source and route of contamination. Currently, the classical bacterial typing methods such as lysozyme typing and serotyping are gradually being replaced by molecular biological methods (Dieckmann et al., 2016). The strategies of pulsed field gel electrophoresis (PFGE) and multilocus sequence typing (MLST) are contributing to global epidemiological and evolutionary studies (Gona et al., 2020). In particular, MLST is an unambiguous procedure for effectively determining bacterial population structure and genealogical assignment based on sequence data of standardized fragments of housekeeping genes (Mammina et al., 2009). However, the procedure of data analysis in large-scale studies requires high cost and time-consuming, limiting the use of MLST. Therefore, novel methods which are amenable for achieving rapid and accurate identification of *K. pneumoniae* typing need to be developed. In this study, based on the MLST typing results, we used SERS spectra for verification and analyzed the fingerprints of different ST types, which showed that the chemometric analysis method was able to distinguish closely related *K. pneumoniae* strains with different ST types based on SERS spectra.

Previous studies have shown that the SERS spectra of different bacteria contain all the information of all molecules in the bacteria. For bacteria, different morphological or physiological characteristics have different molecular basis (Lu et al., 2020). Therefore, it is reasonable to assume that the average SERS spectra can be generated according to the difference in the distribution of characteristic peaks of different bacterial spectra, and bacteria of different species and genera can be easily and rapidly distinguished (Wang et al., 2022). As a proof of concept, we collected the SERS spectra of 16 *K. pneumoniae* strains with unique ST types and measured their SERS spectra. Reproducible SERS spectra (N = 45) were collected for each ST to obtain enough spectra for covering the different morphological and physiological characteristics, simultaneously the average SERS spectra of the different ST types were calculated to avoid the variability of any single spectrum (Fig. 3A). However, the ST types of *K. pneumoniae* were less related, which made it difficult to distinguish the differences in average spectra between different ST types.

Considering the similarity between the average Raman spectra of multiple ST types, Pezzotti et al. (2022) used the linear polynomial expression of the Gauss-Lorentzian function to match the experimental spectra of the minimum scattering based on the average Raman spectra to generate the deconvolution curve of a series of bacteria in the experiment. The experimental results showed that although there were some morphological similarities among seven *Candida auris* significant differences between the deconvoluted spectra could be readily identified. Similarly, we fitted peaks to the SERS spectrum *via* the *Vogit* linear function, which show significant vibrational differences between the different *K. pneumoniae* ST types (Fig. 3B). For example, in the comparison between two bacterial types ST268 and ST866, ST268 has two characteristic peaks at 800–900 cm^−1^, that is, 790 cm^−1^ (cytosine, uracil) (Witkowska et al., 2017) and 850 cm^−1^ (DNA/RNA) (Bandeliuk et al., 2022), respectively. while the two characteristic peaks in the range of 1,650–1,750 cm^−1^ are 1,675 cm^−1^ (C=C and C=O stretching vibrations) (Pezzotti, 2021) and 1,695 cm^−1^ (-C=CA-stretching) (Heidari Torkabadi et al., 2014). The existence of these four spectral deconvolution peaks could be exploited distinguishing ST268 from ST866. Although this method is able to show the “phenotypic difference” among different ST types, it is strongly influenced by the relative intensity of the characteristic peaks of the SERS spectra, which makes it much less useful in practice. Therefore, machine learning algorithms based on advanced statistical methods have been used for subsequent spectral data analysis that greatly improves time efficiency and application potential.

As a supervised clustering analysis algorithm, OPLS-DA is widely applied in the task of distinguishing SERS spectral data (Liu et al., 2023; Tang et al., 2022). Due to the high dimensionality of SERS data, Cheng et al. (2022) found that different leukemia cells could be identified by their intrinsic phenotypic Raman spectra identified *via* the analysis of OPLS-DA algorithm. In order to show the differences between different *K. pneumoniae* ST types and the internal relationship of the same ST type, we used the OPLS-DA algorithm to perform cluster analysis on the spectral data of 16 *K. pneumoniae* ST types (Fig. 4), the result showed that the spectral sample points of different ST types were clustered into different clusters, and the model evaluation indices were R2X = 1.00, R2Y = 0.87, and Q2 = 0.87, indicating that OPLS-DA had a strong ability to distinguish different ST types of SERS spectra. In order to improve the application of machine learning methods in different SERS spectra, and realize the “end-to-end” rapid classification and prediction of spectral data, this study aims to build a spectral identification model suitable for different ST types, and to achieve rapid diagnosis of bacterial typing.

In a clinical diagnostic study on multidrug-resistant *K. pneumoniae*, Lyu et al. (2023) collected 121 strains of *K. pneumoniae* with different resistance profiles, which achieved a predictive accuracy of 99.46% by utilizing convolutional neural network (CNN) combined with attention mechanism. This study confirmed the accuracy and feasibility of SERS spectroscopy for distinguishing *K. pneumoniae* with the assistance of machine learning algorithms. The high sensitivity of the SERS signal and the interference of factors such as the coffee ring effect during sample preparation could lead to large differences between Raman spectra of isotypes (Koya et al., 2019), which will further affect the performance of the model. Therefore, in order to attain a classification model that exhibits similar high accuracy and stable performance, the parameter search of the machine learning algorithm is very important. The *GridSearchCV* method originated from scikit-learn library is able to rapidly search for the optimal hyperparameters (Gao et al., 2022; Lei et al., 2022). In a recent study performed by Wang et al. (2022) the *GridSearchCV* method was utilized to optimize the parameters of three machine learning models when analyzing the SERS spectral data of 30 bacteria strains from 9 different genera isolated from clinical samples. In another study of Raman fingerprint of spoilage fungi, Guo et al. (2021) used a grid search to optimize the hyperparameters of the model and showed the process with a grid gradient and the optimized values. In this study, for the six different machine learning algorithm models used in this study, we set the parameter range of each model separately, and used the grid search gradient map (Fig. 5) to show the fitting process of each machine learning model. From the model fitting results, it can be found that the SVM algorithm (Fig. 5E) maintains 100% identification accuracy in all parameter combinations, indicating that the SVM model can be well applied to the spectral data analysis of different ST types. In contrast to previous studies, Ciloglu et al. (2022) employed a non-linear autoencoder algorithm to extract spectral features when using the SVM algorithm to differentiate colistin-resistant and susceptible strains of *K. pneumoniae*. Their autoencoder-SVM model achieved an accuracy of 94%. However, in the course of this study, it was discovered that the feature extraction process was unnecessary, as the SVM model alone yielded satisfactory results. The best combination of parameters (Table S1) fitted to each model is fed into the algorithm for model training, and the test set samples are used to test the real application performance of each algorithm. Different evaluation metrics are often used to measure the performance of machine learning (Ma et al., 2023; Tang et al., 2022). In this study, we comprehensively considered the performance of different algorithms in all indicators (Table 2), and found that the SVM algorithm scored 100% in all indicators, and the model fitting time was relatively short. In sum, our results show that SVM is an efficient and stable algorithm suitable for ST typing of *K. pneumoniae*, and has potential application for rapid tracing of the spread and control of *K. pneumoniae* in hospitals and communities.

## Conclusions

*K. pneumoniae* is a major public health concern worldwide due to its high mortality rate in clinical settings. Rapid and accurate identification and discrimination of different ST types of *K. pneumoniae* is crucial for monitoring and controlling the spread of *K. pneumoniae*. However, the complexity and high cost of traditional methods make efficient and cheap bacterial typing methods urgently needed. This study explored the performance of SERS technology, combining it with multiple advanced machine learning algorithms, for the identification of 16 different ST-typed *K. pneumoniae*. Experimental results show that Raman spectroscopy is sufficient to obtain high-quality bacterial SERS spectra in clinical laboratories, and that intrinsic differences between different ST typings are revealed by averaging SERS spectra and spectral deconvolution. Through OPLS-DA analysis, it is found that different types of bacterial spectral sample points can be automatically divided into different clusters. Comparing the performance of different machine learning models, the SVM algorithm can accurately classify and predict each type of *K. pneumoniae*, which is consistent with the MLST results. In summary, this study confirms that SERS technology combined with machine learning algorithm can accurately predict the ST types of different *K. pneumoniae*, and has the potential for clinical application with low cost, high speed and high accuracy, and lays the foundation for SERS technology in hospital and community infection detection.

## Supplemental Information

10.7717/peerj.16161/supp-1 Supplemental Information 1 The Best Combination of hyperparameter for Different Machine Learning Algorithms.

10.7717/peerj.16161/supp-2 Supplemental Information 2 Band assignments of characteristic peaks to potential metabolites in SERS spectra for *K. pneumoniae*.

10.7717/peerj.16161/supp-3 Supplemental Information 3 Raw measurements.

## Additional Information and Declarations

### DNA Deposition

The following information was supplied regarding the deposition of DNA sequences: The genome sequencing and assembly is available at NCBI: PRJNA960686.

### Data Availability

The following information was supplied regarding data availability: The raw measurements are available in the Supplemental File.
