# Supplementary material for: Classification and prediction of Klebsiella pneumoniae strains with different MLST allelic profiles via SERS spectral analysis
Source: PeerJ. 2023 Sep 25;11:e16161. doi: 10.7717/peerj.16161 (PMC10538299; doi:10.7717/peerj.16161)
Supplement: Supplemental Information 1 [file peerj-11-16161-s001.docx]

**Supplementary Table S1** The Best Combination of hyperparameter for Different Machine Learning Algorithms

| **Algorithms** | **Function** | **Parameter Range** | **Optimum Parameter** |
| --- | --- | --- | --- |
| **AdaBoost** | AdaBoostClassifier | **learning_rate** = [0.1, 1, 0.01, 0.001],  **n_estimators** = [50, 60, 70, 80, 90, 100, 110, 120, 130, 140, 150, 160, 170, 180] | **learning_rate** = 0.01, **n_estimators** = 60 |
| **Decision Tree** | DecisionTreeClassifier | **criterion** = ['gini', 'entropy'],  **max_depth** = range (1, 30), **max_features** = [21, 22, 23, 24, 25, 26, 28, 29, 30, 'auto'] | **criterion** = 'gini',  **max_depth** = 21,  **max_features** = 24 |
| **Quadratic**  **Discriminant** | QuadraticDiscriminantAnalysis | **reg_param =** np.linspace(0, 1, 10),  **tol =** [0.0001, 0.001, 0.01, 0.1, 1] | **reg_param =** 1.0,  **tol =** 0.0001 |
| **Random Forest** | RandomForestClassifier | **Criterion** = ['gini', 'entropy'],  **max_depth** = range (1, 10),  **n_estimators** = [50, 60, 70, 80, 90, 100, 110, 120, 130, 140, 150, 160, 170, 180] | **criterion** = 'entropy', **max_depth** = 8, **n_estimators** = 150 |
| **SVM** | SVC | **Cs =** [0.0001, 0.001, 0.01, 0.1, 1, 2, 3, 4, 5, 10],  **gamma =** [0.0001, 0.001, 0.01, 0.1, 1],  kernel = ['rbf', ' linear '] | **Cs =** 0.01,  **gamma =** 0.01,  **kernel =** 'linear' |
| **XGBoost** | XGBClassifier | **n_estimators =** [50, 60, 70, 80, 90, 100, 110, 120, 130, 140, 150, 160, 170, 180],  **learning_ rate =** [0.1, 1, 0.01, 0.001] | **Estimators** = 70, **learning_rate** = 0.01 |
