# Supplementary material for: Classification and prediction of Klebsiella pneumoniae strains with different MLST allelic profiles via SERS spectral analysis
Source: PeerJ. 2023 Sep 25;11:e16161. doi: 10.7717/peerj.16161 (PMC10538299; doi:10.7717/peerj.16161)
Supplement: Supplemental Information 2 [file peerj-11-16161-s002.docx]

**Supplementary Table S1** Band assignments of characteristic peaks to potential metabolites in SERS spectra for *K. pneumoniae*.

| **Wavenumber (cm^-1^)** | **Band Assignment** | **Ref.** |
| --- | --- | --- |
| 558 | Carbohydrate, β(O-C-O) | [1, 2] |
| 626 | aromatic ring skeletal | [3] |
| 652/653/655/658 | C-S str, C-C twist of proteins tyrosine, (COO2) in amino acids, δdeformation (guanine), ν (C-S) in cysteine, guanine | [2, 4] |
| 726/733 | adenine, glycosidic ring mode | [3, 5, 6] |
| 853/854 | C–C stretch in 1,4 glycosidic link | [2, 3] |
| 957/961 | γ(CN), Membrane phospholipids | [2, 3, 5] |
| 1023 | A ring stretching, or (C-H) deformation | [2] |
| 1092/1094/1095, 1096/1098 | Protein, DNA backbone-phosphate backbone | [3, 7] |
| 1134/1137 | γ streching(COC), ring breathing | [2, 3] |
| 1220 | Amide III, adenine, polyadenine and DNA | [3] |
| 1240/1243 | Amide III β-sheet, Amide I | [2, 6] |
| 1326/1330/1335 | υ(NH2) adenine, polyadenine, DNA | [3] |
| 1448 | C-H_2_ scissoring | [6] |
| 1455/1457/1460  1463/1464/1465 | δ(CH2) saturated lipids | [3, 5] |
| 1538 | amide II, υ(CN), γ(NH) | [3] |
| 1576/1579/1580 1585/1589/1590 | Tyrosine (proteins) | [2] |
| 1676 | Amide I, protein | [2] |
| 1684/1685 | Amide Ⅰ | [8] |

1. Lyu JW, Zhang XD, Tang JW, Zhao YH, Liu SL, Zhao Y, Zhang N, Wang D, Ye L, Chen XL *et al*: **Rapid Prediction of Multidrug-Resistant Klebsiella pneumoniae through Deep Learning Analysis of SERS Spectra**. *Microbiol Spectr* 2023:e0412622.

2. Mungroo NA, Oliveira G, Neethirajan S: **SERS based point-of-care detection of food-borne pathogens**. *Microchimica Acta* 2016, **183**(2):697-707.

3. Liu Y, Zhou H, Hu Z, Yu G, Yang D, Zhao J: **Label and label-free based surface-enhanced Raman scattering for pathogen bacteria detection: A review**. *Biosens Bioelectron* 2017, **94**:131-140.

4. Nowicka AB, Czaplicka M, Szymborski T, Kaminska A: **Combined negative dielectrophoresis with a flexible SERS platform as a novel strategy for rapid detection and identification of bacteria**. *Anal Bioanal Chem* 2021, **413**(7):2007-2020.

5. Zhou X, Hu Z, Yang D, Xie S, Jiang Z, Niessner R, Haisch C, Zhou H, Sun P: **Bacteria Detection: From Powerful SERS to Its Advanced Compatible Techniques**. *Adv Sci (Weinh)* 2020, **7**(23):2001739.

6. Cheong Y, Kim YJ, Kang H, Choi S, Lee HJ: **Rapid label-free identification of Klebsiella pneumoniae antibiotic resistant strains by the drop-coating deposition surface-enhanced Raman scattering method**. *Spectrochim Acta A Mol Biomol Spectrosc* 2017, **183**:53-59.

7. Lu X, Samuelson DR, Xu Y, Zhang H, Wang S, Rasco BA, Xu J, Konkel ME: **Detecting and tracking nosocomial methicillin-resistant Staphylococcus aureus using a microfluidic SERS biosensor**. *Anal Chem* 2013, **85**(4):2320-2327.

8. Shang L, Xu L, Wang Y, Liu K, Liang P, Zhou S, Chen F, Peng H, Zhou C, Lu Z *et al*: **Rapid detection of beer spoilage bacteria based on label-free SERS technology**. *Anal Methods* 2022, **14**(48):5056-5064.
